# Did our pharmacological strategy for COVID‐19 fail?

**DOI:** 10.1002/prp2.866

**Published:** 2021-10-27

**Authors:** Jennifer H. Martin, Michelle Baddeley, Richard Head

**Affiliations:** ^1^ University of Newcastle Hunter Medical Research Institute New Lambton New South Wales Australia; ^2^ University of Technology Sydney Ultimo New South Wales Australia; ^3^ University of South Australia Clinical & Health Sciences Adelaide South Australia Australia

**Keywords:** cognitive bias, COVID‐19, observational study, renin angiotensin system, statins

We are now 20 months into the pandemic. One could assume that a global community of well‐funded medical researchers and discovery scientists armed with descriptive clinical information would have discovered a wealth of treatments by now. Yet for ***mainstream and global treatment***, we really have only one 60‐year‐old therapeutic family (the steroids) which only marginally improves outcomes for people who require oxygen treatment.^1^, ^2^ This is barely better than our response to influenza in 1918 yet over the last 100 years we have made significant strides in understanding viruses and the body's physiological response to the resulting virus‐induced inflammation which is a major predictor of morbidity and mortality.

And still, despite the presence of a variety of antiviral therapies and anti‐inflammatory cytokine antibodies, prohibitively expensive for the majority of the global population, and with at best, a reduction in viral load or only small clinical efficacy for some.^3^ it would be hard to say we have a robust therapeutic pipeline for the remainder of this and for future pandemics. Thus, despite vaccines, which have vastly improved outcomes for people who live in high‐income countries, therapeutic options are still needed. Not everyone can be vaccinated and some vaccines are more effective than others (note that this also applies to antibody therapies). Virus mutations represent an ongoing challenge. A **strategy** for pandemic pharmacology and therapeutics development is thus vital because months of time and hundreds of thousands of lives have already been lost without treatment options available. The Economist over 4 months ago estimated that there were up to ***13 million*** excess deaths from COVID‐19.^4^ Furthermore, the therapies “chosen” by many funding bodies and health organizations, such as hydroxychloroquine, for example, have caused worse morbidity, distracted the research and funding community from trialing other therapies that have a more clinically relevant pharmacological justification. Moreover, patients who require some of these drugs for rheumatological and antimalarial diseases have subsequently had difficulty obtaining them. Overall, these activities represent a huge opportunity cost for patients.^5^

In distinction, a variety of strategies and solutions have been made available by pharmacology and therapeutics groups to help guide a global priority list for rapid clinical trials.^6^ In early 2020 several pharmacologists and physicians acknowledged that a “buying time” strategy was needed for this pandemic.^7^ This included the rapid clinical trialing of therapies that observational data (clinical and routine administrative) had already appeared to show morbidity and mortality benefits, and the use of propensity scoring to overcome the issues of confounding in the observational data. Randomized clinical trials (RCTs) were known to be the optimal type of clinical study, yet with a raging pandemic, time and money were of the essence. Collecting observational data was a more realistic approach; the budesonide study provides a good example of how it can be done.^2^ This study allowed a drug to be used whilst waiting for a more definitive RCT to be undertaken to quantify the size of the benefit.

Yet publication of similarly helpful work from across the world proved difficult. Pearson has outlined the wastage that has occurred due to poorly thought our choices of therapeutics and inappropriate sample sizes in COVID trials.^8^ Several projects have shown no or minimal benefit or only harms. Many of these studies have been too underpowered to answer the question of interest and drugs were studied that were not based on the clinical physiology of COVID nor the clinical pharmacology of the drugs themselves. This report summarizes numerous trials of putative therapeutics that expended significant research dollars without demonstrating meaningful benefits.^8^

Another large difference in a pandemic compared to non‐pandemic times is that the settings of complex and variable unmet health needs are required using methods in which randomized control trials are either unfeasible, impractical, or unethical.^8^ Others have noted that even if the limitations of RCTs in a pandemic can be overcome, those who would or could fund or conduct RCTs fail to present a unified plan.^9^ Many of the hundreds of COVID‐19 trials registered on ClinicalTrials.gov intend to test a wide array of interventions but few have been shown to be effective, have taken a long time to complete and are prohibitively expensive for most research teams.

For example, both observational studies and also RCTs (although underpowered for mortality benefit) have shown the benefit of using commonly used drugs that block the renin–angiotensin–aldosterone system (RAS) in virus diseases, including influenza, Ebola, and others.^10^ Observational data for statins have shown similar results.^11^, ^12^ Now we have more observational evidence with respect to the utility of these inexpensive and relatively safe therapies in COVID, including their combination, in real‐world patients, using death as an unbiased endpoint.^13^ A group of investigators in Belgium has recently published an observational study of the impact of RAS inhibitors and statins (and their combination) on 28‐day mortality in 959 COVID‐19 hospitalized patients. Using propensity scores, all treated and untreated patients were matched. For combination treatment with statins plus ACEIs/ARBs, the adjusted odds ratio (OR) for 28‐day hospital mortality was 0.33 (95% CI 0.17–0.69; *p* = 0.002, SMD = 0.22). For treatment with statins alone and ACEIs/ARBs alone the adjusted ORs were 0.56 (95% CI 0.39–0.93, *p* = 0.024, SMD = 0.08) and 0.52 (95% CI 0.23–1.17, *p* = 0.11, SMD = 0.006), respectively. These results strongly suggest that patients treated in‐hospital with statins in combination with ACEIs/ARBs experienced a threefold reduction in the odds of 28‐day hospital mortality. It is important to note that these drugs are known to be safe when used in patients with critical illness, are produced as inexpensive generics, and can be found on the shelves of every hospital. Their dose–response ranges for all toxicities and efficacies for cardiovascular diseases are known to all practicing physicians.

Yet the global platform trials for COVID‐19 treatments have still not chosen these drugs for study nor have they been recommended for physician use in patient care, although such recommendations could easily be justified based on the magnitude of their observed benefits, costs, and easy availability. We have previously highlighted the compelling need to explore and where possible align the known pathophysiology of the infection with repurposed agents of this type,^6^, ^7^ albeit acknowledging that drug repurposing as a therapeutic strategy has not been a great success to date, perhaps due to incomplete understanding to the targeted disease state coupled with imperfect knowledge of the interventional mechanism of action.

## 1 SO: WHAT THERAPEUTICS STRATEGIES HAVE WORKED?

In looking at what has worked, the answer is clear—success has been achieved when there has been a solid biological and pharmacologically based rationale and knowledge of how to transfer this information to sick patients. The work of the RECOVERY investigators focused on treating the host with dexamethasone to dampen the inflammatory and pro‐angiotensin II response to COVID‐19.^1^

It is notable that what is known about common therapies such as RAS agents and statins has not been translated into clinical practice. A descriptive review in this edition of the Journal^14^ shows positive data for the use of statins based on observational data. Five journals rejected the manuscript of this report despite the urgent need for effective COVID‐19 treatment and the large amount of evidence showing its benefits (*personal communication David Fedson*).

## 2 WHAT IS THE PROBLEM?

One possible explanation for the problems in leading a pharmacology and therapeutics strategy to date, is bias. The pre‐pandemic method of allocating grant funding and favoring only RCTs, of having the time to have all the certainty that is needed before access to therapy may also be a bias. It is well known that behaviors and cognitive bias in belief systems contribute to the inability to adopt best practices. And although not disconnected, it is interesting to independently analyze both the effects these biases have on (1) decision makers and scientists and (2) individuals whose biases are reinforced by sensationalist stories, news media, and the internet.

The COVID‐19 pandemic has verified well‐established findings from behavioral economics about how availability and affect heuristics can lead individuals to overestimate the risks of unlikely events when the information they draw upon is distorted by vivid and emotive stories.^15^, ^16^, ^17^ Their biases can be magnified by sensationalist news that may have more salience than objective scientific analyses. In the presence of herding and other social influences, these biases are hard to contain within small groups of people, especially when peer pressure accelerates the spread of misinformation.^18^ An individual who is not skilled in risk‐based decision making or not privy to relevant information can find it difficult to make the right decision.

## 3 WHAT IS THE SOLUTION?

**FIGURE 1:**
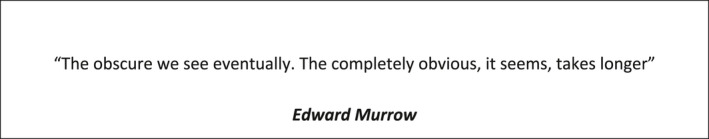
A way of thinking to enable a solution

We believe the solution is embodied in the Murrow quote (Figure 1). The completely obvious in the Murrow quote is well illustrated by published observational findings indicating that COVID‐19 patients admitted to hospital with asthma and chronic obstructive pulmonary disease were significantly under‐represented.^2^ This group then explored a physiological and pharmacological potential beneficial role of inhaled glucocorticoids in this pandemic and conducted a randomized Phase II RCT that demonstrated a clinical benefit of inhaled budesonide. This approach meant that doctors could use inhaled budesonide to treat COVID‐19 patients until comparative efficacy and safety data become available. The obvious is the importance of the original observational studies and data linkage, informing the subsequent research, together with an understanding of how to acknowledge, interpret and manage the bias and lack of certainty in these measurements.

The solution may also not be a comparison of the relative merits of RCTs compared with observational studies but the power of combining both in sequence to enable speed and guidance. The two methods must be used in concert when one seeks knowledge that is useful. This is especially so for treatment studies that cover only a short time frame such as 7 days post‐admission to ICU with COVID. In designing observational studies, it is possible to use methods such as propensity scoring to generate treatment and comparison groups that are comparable. High‐quality sensitivity analyses can show the low likelihood that confounding has compromised the results. The combination of observational studies and RCTs can be further bolstered by the known pathophysiology induced by the pandemic pathogen^6^ as was seen within the budesonide study.^2^

As additional support for not waiting for RCT data in a pandemic, the observational findings from the Belgian group are aligned with those of other studies of in‐hospital treatment with statins and drugs that inhibit the RAS.^19^, ^20^, ^21^ Considered together, these findings are consistent with criteria we have published using RAS inhibitory drugs widely available in worldwide formularies and essential medicines lists.^6^ The use of these drugs also aligns with the well‐known anticoagulant, and anti‐inflammatory effects of both statins and RAS agents.^22^, ^23^ Consistent with findings of the Belgian group, Duarte et al. have suggested that the ARB telmisartan in high doses could reduce morbidity and mortality in hospitalized patients infected with SARS‐CoV2.^24^

It seems odd that such clearly safe, cheap, and apparently successful treatment strategy that might have an impact on the thousands of deaths per day worldwide has not been given more prominence. Accordingly, we believe this highlights the need for greater strategic calibration of the utility of existing therapeutics and pharmaceutical sciences in a pandemic. The urgent need in a pandemic is to better drive the interface between information technologies, administrative data, pharmaceutical sciences, and the integration of observational studies and randomized controlled trials.

Finally, we can learn a great deal by exploring what at first seems obscure but is really self‐evident. System‐based analysis can lead to meaningful action in a pandemic. It can direct us to explore the self‐evident; to better understand and address cognitive biases and to exploit the portfolio of existing therapeutics that will give us enough time to implement vaccination programs. Approaches that are suitable in normal settings rarely translate effectively to a crisis environment. The need for a logically driven, simple and yet coordinated approach to meet unmet needs is clear. It is notable that the major contribution to the therapeutic strategy in this pandemic—dexamethasone—was noted by the authors to be achieved by asking a simple clinical question and providing a well‐designed and appropriately powered RCT.

While the obscure and self‐evident are key we would do well to recognize the wisdom of George Orwell who wrote “to see what is in front of one's nose needs a constant struggle”.^1^

## ENDNOTE

^1^

https://orwell.ru/library/articles/nose/english/e_nose
